# Lipid Profile, Apolipoproteins, and Impulsivity in First Episode Mania

**DOI:** 10.7759/cureus.94335

**Published:** 2025-10-11

**Authors:** Rasmita Behera, Sarada P Swain, Pratima Sahu, Saffalya Nayak, Debjyoti Mohapatra

**Affiliations:** 1 Psychiatry, Government Medical College and Hospital, Kandhamal, IND; 2 Psychiatry, Mental Health Institute, Center of Excellence, and Sriram Chandra Bhanja (SCB) Medical College and Hospital, Cuttack, IND; 3 Biochemistry, Sriram Chandra Bhanja (SCB) Medical College and Hospital, Cuttack, IND; 4 Community Medicine, Shri Jagannath Medical College and Hospital, Puri, IND

**Keywords:** apolipoproteins, bipolar disorder, cholesterol, impulsivity, lipid profile, mania

## Abstract

Background: Mania, a core feature of bipolar disorder, is characterized by impulsivity, hyperactivity, and mood disturbances. Impulsivity has been linked to lipid metabolism, particularly cholesterol and apolipoproteins. This study investigates the relationship between lipid profile, apolipoprotein A1 (ApoA1) and apolipoprotein B (ApoB), and impulsivity in first-episode mania patients.

Methods: A case-control study was conducted at Sriram Chandra Bhanja (SCB) Medical College, Cuttack, involving 60 patients with first-episode mania and 60 age-matched healthy controls. Lipid parameters, including total cholesterol (TC), triglycerides (TG), high-density lipoprotein (HDL), low-density lipoprotein (LDL), very-low-density lipoprotein (VLDL), ApoA1, and ApoB, were measured. Impulsivity was assessed using the Barratt Impulsiveness Scale (BIS-11). Independent samples t-tests and Pearson's correlation were used for statistical analysis.

Results: Mania patients had significantly lower TC (156.58 ± 14.00 mg/dL vs. 175.93 ± 23.59 mg/dL, p < 0.001), LDL (75.00 ± 9.24 mg/dL vs. 83.58 ± 16.86 mg/dL, p = 0.001), and TG (74.03 ± 11.94 mg/dL vs. 96.43 ± 29.48 mg/dL, p < 0.001) compared to controls. ApoB levels were higher in mania patients (795.95 ± 725.44 mg/dL vs. 549.53 ± 796.67 mg/dL, p = 0.079), though not statistically significant. BIS-11 scores negatively correlated with cholesterol levels, particularly TC and LDL, suggesting an association between hypercholesterolemia and increased impulsivity.

Conclusion: Lower cholesterol levels, particularly LDL, are significantly associated with impulsivity in first-episode mania patients. These findings highlight the potential role of lipid metabolism in psychiatric disorders and suggest lipid monitoring in high-risk individuals.

## Introduction

Mania is an episodic presentation in bipolar disorder (BD) and is characterized by elevated mood, increased energy, impulsivity, and hyperactivity, often resulting in impaired judgment and psychosocial dysfunction [1]. Among these symptoms, impulsivity is a core feature that contributes to high-risk behaviors, aggression, and suicidality [2]. Impulsivity is described as actions that are poorly conceived, prematurely expressed, unduly risky, or inappropriate to the situation, often resulting in undesirable consequences. Impulsivity, in relation to affective disorders, has been studied extensively, and a significantly higher level of impulsivity has been described in affective disorders compared with the normal population [3,4]. Researchers have also found an increased impulsivity in euthymic bipolar patients, though the acute neurochemical changes in brain probably lead to a particularly overt manifestation of impulsivity during manic episodes. The neurobiological basis of impulsivity is complex and has been associated with serotonergic dysfunction, lipid metabolism, and neurotransmitter deregulation [4,5]. Emerging evidence suggests that cholesterol plays a crucial role in neuronal function, influencing synaptic plasticity and neurotransmission, particularly serotonin (5-HT) function. Deficient central serotonergic transmission has been proposed as a biological substrate for impulsivity; and a number of studies in past have suggested serum cholesterol to be a surrogate marker for the same and demonstrated a correlation between serum cholesterol and various measures of impulsivity. Cholesterol depletion has also been found to result in impaired functioning of 5-HT1A and 5-HT7 receptors. Cholesterol is also a major component of lipid rafts, which are of significance in synaptic function and thus depletion of cholesterol has been shown to have diffuse effects on not only serotonergic functioning, but also on other neurotransmitter systems, including the excitatory amino acid transport and gamma-aminobutyric acid transmission [5,6]. 

The patients presenting with low cholesterol, increased impulsivity, and mood symptoms require clinical attention and surveillance because of high-risk behavior [5,6]. In addition, apolipoproteins, particularly apolipoprotein A1 (ApoA1) and apolipoprotein B (ApoB), play a role in lipid transport and inflammation implicated in mood disorders. ApoA1 (a major component of high-density lipoprotein (HDL)) and ApoB (a major component of low-density lipoprotein (LDL)) are thought to be involved in the inflammatory processes and neuroprogression associated with bipolar disorder [5-7]. Several studies have found that bipolar patients, particularly those experiencing mania, tend to have altered lipid profiles, characterized by lower total cholesterol (TC) and LDL levels, which are associated with increased impulsivity [5,8]. Again, most of the studies investigating the relationship between cholesterol and impulsivity have been done in Western countries, where the diet/lifestyle is different from Indian population, which justifies the study [5,8,9].

The aim of the study was to explore the relationship between lipid profiles, apolipoproteins (ApoA1 and ApoB), and impulsivity in first-episode mania in developing countries. By assessing apolipoprotein levels, which serve as lipid transporters and regulate lipid concentrations, this research represents a novel approach in the context of first-episode mania. A better insight into these associations may enhance understanding of the biological mechanisms underlying impulsivity in bipolar disorder and facilitate the identification of metabolic biomarkers for early detection and targeted interventions. We hypothesize that lower TC and LDL would be associated with higher impulsivity in first-episode mania.

## Materials and methods

A hospital-based case-control study was conducted from December 2021 to November 2022 at the Mental Health Institute (Center of Excellence), in partnership with the Postgraduate Department of Biochemistry at Sriram Chandra Bhanja (SCB) Medical College and Hospital, a tertiary care institution located in Cuttack, Odisha, India. The overall number of subjects who participated in the study was 120, where 60 patients with first-episode mania were selected in our study and 60 controls who were matched for age (±5), gender (male/female) and geographical location (rural/urban) were selected as the control group. The ICD-10 DCR (World Health Organization, 1993) criteria were used to assist in the diagnosis of patients [10]. All participants gave their written informed consent. In manic patients, informed consent was taken from their nominated representatives and caregivers.

Participants were required to be at least 18 years old, drug-naïve, or drug-free for a minimum of two weeks (for oral drugs) or four weeks (for depot formulations) before they could participate in the study. Those individuals who were excluded from the study were those who had a history of abnormal cholesterol levels, neurological diseases, or chronic medical conditions that affected lipid metabolism. The body mass index (BMI) of all participants was recorded. The Young Mania Rating Scale (YMRS) was used to measure the severity of manic symptoms among the study population [11]. A clinical examination that measured impulsivity was the Barratt Impulsiveness Scale (BIS-11), which was included in the evaluation. BIS-11 is the most commonly used self-report measure for assessing impulsivity in both clinical and research settings. There are 30 personal statements in the BIS-11, as designed to assess general impulsiveness taking into account the multi-factorial nature of the construct. Items are rated from 1 (absent) to 4 (most extreme), and scores range from 30 to 120 [12].

An overnight fast of twelve hours was followed by the collection of blood samples, and the enzymatic colorimetric method was utilized to examine lipid parameters. These parameters included total cholesterol (TC), triglycerides (TG), high-density lipoprotein (HDL), low-density lipoprotein (LDL), and very-low-density lipoprotein (VLDL). An immunoturbidimetric analysis was performed in order to determine the levels of apolipoprotein A1 (ApoA1) and apolipoprotein B (ApoB).

SPSS version 23 (IBM Corp., Armonk, New York) was used to carry out the statistical analysis [13]. Continuous variables were expressed as mean ± standard deviation (SD), while categorical variables were presented as frequencies and percentages. Pearson correlation was applied to evaluate the relationships between impulsivity scores and lipid levels, and between-group comparisons were conducted using an independent t-test. The Institutional Ethics Committee (IEC) of SCB Medical College approved ethical permission (IEC no: 935/01.12.2021), and all procedures were conducted in accordance with the Declaration of Helsinki.

## Results

Table 1 summarizes the demographic and clinical characteristics of the study participants. Among the study population, 61.7% of participants have a moderate level of symptoms, and 38.3% have a severe level of symptoms as per YMRS criteria. Demographic and clinical characteristics between 60 mania patients and 60 controls are compared in Table 1. The groups were matched for gender, age, and geographical region, with no significant differences in these variables. No significant differences were observed between the groups in terms of religion, education, marital status, monthly income, family type, or BMI. However, a significantly higher proportion of mania patients had a family history of psychiatric illness (18.3% vs. 3.3%, p = 0.0188). Mania patients also showed markedly higher impulsivity scores across all Barratt Impulsiveness Scale (BIS-11) domains (attentional, motor, non-planning, and total scores) compared to controls (p < 0.001). Among mania patients, 65% were categorized as highly impulsive. There was no significant difference in the mean age between mania patients (30.83 ± 7.16 years) and healthy controls (29.28 ± 12.01 years; p = 0.392). However, the body mass index (BMI) was significantly higher in mania patients compared to controls (2.25 ± 0.47 vs. 2.03 ± 0.49; p = 0.015 (Table 1).

**Table 1:** Comparison of demographic and clinical characteristics between mania patients and controls. Dashes (-) indicate data not applicable for chi-square or continuous data. Chi-square test has been used for categorical variables, and independent samples t-test for numerical variables. BMI: body mass index, YMRS: Young Mania Rating Scale, BIS-11: Barratt Impulsiveness Scale-11.

Characteristic	Category	Mania (N = 60)	Control (N = 60)	p-value
Gender	Male	46 (76.7%)	46 (76.7%)	1.0000
	Female	14 (23.3%)	14 (23.3%)	
Mean age	Mean age	30.83 ± 7.16	29.28 ± 12.01	0.392
Religion	Hindu	45 (75.0%)	41 (68.3%)	0.5434
	Others	15 (25.0%)	19 (31.7%)	
Education	Primary	31 (51.7%)	34 (56.7%)	0.2432
	High school	26 (43.3%)	19 (31.7%)	
	Intermediate or above	3 (5.0%)	7 (11.7%)	
Marital status	Married	35 (58.3%)	32 (53.3%)	0.7131
	Unmarried	25 (41.7%)	28 (46.7%)	
Monthly income (₹)	<10,000	51 (85.0%)	48 (80.0%)	0.6309
	>10,000	9 (15.0%)	12 (20.0%)	
Family type	Nuclear	11 (18.3%)	17 (28.3%)	0.2805
	Extended	49 (81.7%)	43 (71.7%)	
Geographical region	Rural	54 (90.0%)	54 (90.0%)	1.0000
	Urban	6 (10.0%)	6 (10.0%)	
Drug status	Free	18 (30.0%)	0 (0.0%)	-
	Naïve	42 (70.0%)	60 (100.0%)	
Significant family history	Present	11 (18.3%)	2 (3.3%)	0.0188
	Absent	49 (81.7%)	58 (96.7%)	
BMI (kg/m²)	18.5–22.9	44 (73.3%)	46 (76.7%)	0.1869
	23–24.9	16 (26.7%)	14 (13.3%)	
YMRS mania severity	Severe	23 (38.3%)	0 (0.0%)	-
	Moderate	37 (61.7%)	0 (0.0%)	
	No symptom	0 (0.0%)	60 (100%)	
BIS-11 domain-specific and total score	Attentional score	22.05 ± 3.12	18.03 ± 2.49	<0.001
	Motor score	26.35 ± 3.22	16.22 ± 2.29	<0.001
	Non-planning score	25.50 ± 2.86	15.75 ± 2.07	<0.001
	Total score	73.90 ± 6.57	50 ± 3.16	<0.001
BIS-11 score categorization	Highly impulsive	39 (65.0%)	0 (0.0%)	<0.001
	Normal impulsive	21 (35.0%)	18 (30.0%)	
	Extremely over-controlled	0 (0.0%)	42 (70.0%)	

**Figure 1:**
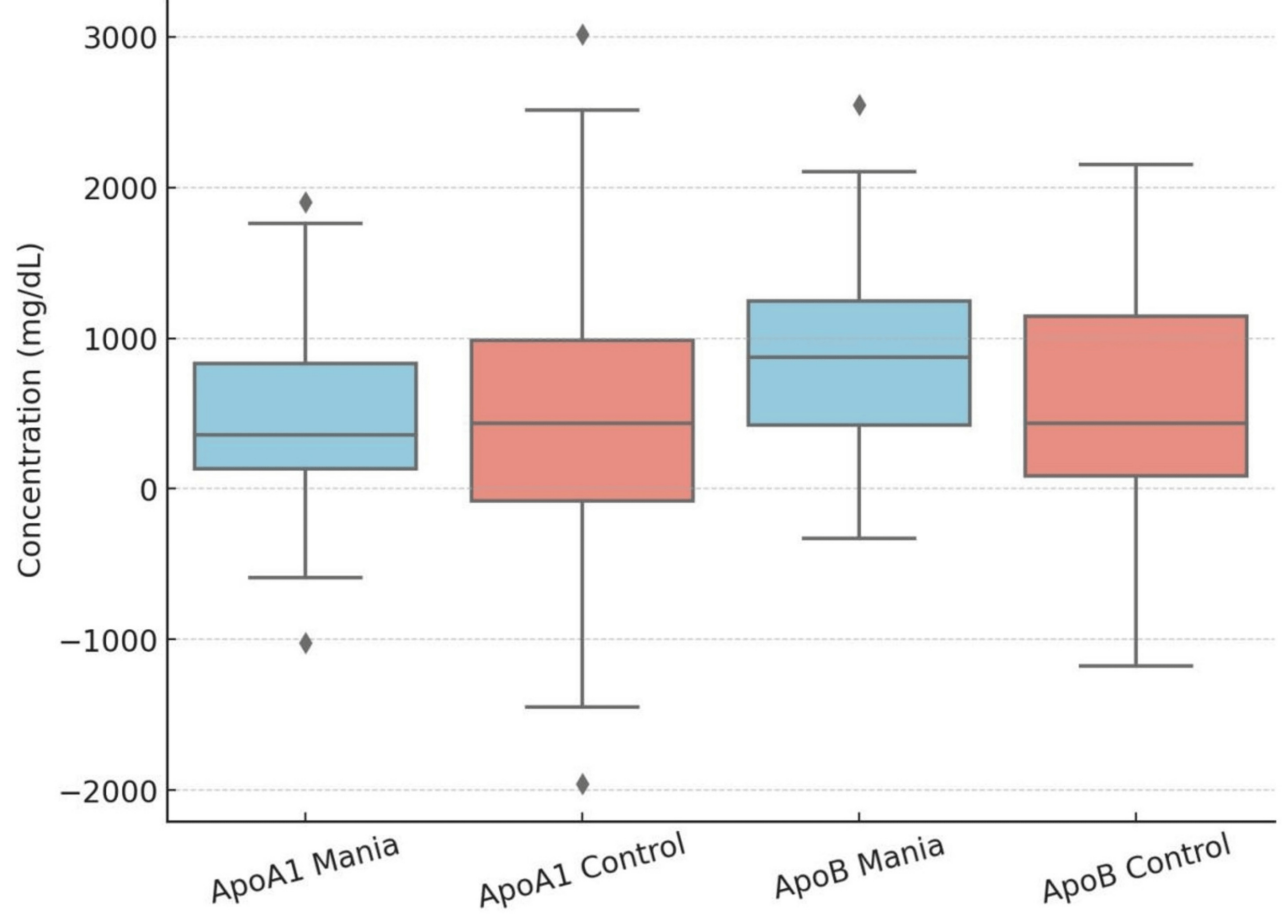
Box-whisker plot comparing Apo A1 and Apo B levels in mania and control groups. ApoA1: apolipoprotein A1; ApoB: apolipoprotein B.

Table 2 shows the comparison of various lipid parameters of first-episode mania patients with those of controls. Mania patients demonstrated significantly reduced levels of total cholesterol (156.58 ± 14.00 mg/dL vs. 175.93 ± 23.59 mg/dL; p < 0.001), triglycerides (74.03 ± 11.94 mg/dL vs. 96.43 ± 29.48 mg/dL; p < 0.001), low-density lipoprotein (LDL) cholesterol (75.00 ± 9.24 mg/dL vs. 83.58 ± 16.86 mg/dL; p = 0.001), and very-low-density lipoprotein (VLDL) cholesterol (25.05 ± 2.84 mg/dL vs. 26.48 ± 3.67 mg/dL; p = 0.018) compared to controls. Although high-density lipoprotein (HDL) cholesterol levels were higher in mania patients (49.10 ± 5.85 mg/dL vs. 47.02 ± 6.55 mg/dL), but the difference was not significant (p = 0.069). No significant differences were observed between groups in apolipoprotein A1 (Apo A1) (p = 0.653) or apolipoprotein B (ApoB) (p = 0.079) levels (Table 2, Figure 1). 

**Table 2:** Comparison of lipid parameters between first-episode mania patients and healthy controls. Independent samples t-test was used to calculate the p-values. HDL: high-density lipoprotein, LDL: low-density lipoprotein, VLDL: very-low-density lipoprotein, ApoA1: apolipoprotein A1, ApoB: apolipoprotein B.

Characteristic/parameter	Mania patients (mean ± SD)	Controls (mean ± SD)	p-value
Total cholesterol (mg/dL)	156.58 ± 14.00	175.93 ± 23.59	<0.001
Triglycerides (mg/dL)	74.03 ± 11.94	96.43 ± 29.48	<0.001
HDL (mg/dL)	49.10 ± 5.85	47.02 ± 6.55	0.069
LDL (mg/dL)	75.00 ± 9.24	83.58 ± 16.86	0.001
VLDL (mg/dL)	25.05 ± 2.84	26.48 ± 3.67	0.018
ApoA1 (mg/dL)	537.45 ± 661.58	471.83 ± 911.57	0.653
ApoB (mg/dL)	795.95 ± 725.44	549.53 ± 796.67	0.079

**Figure 2:**
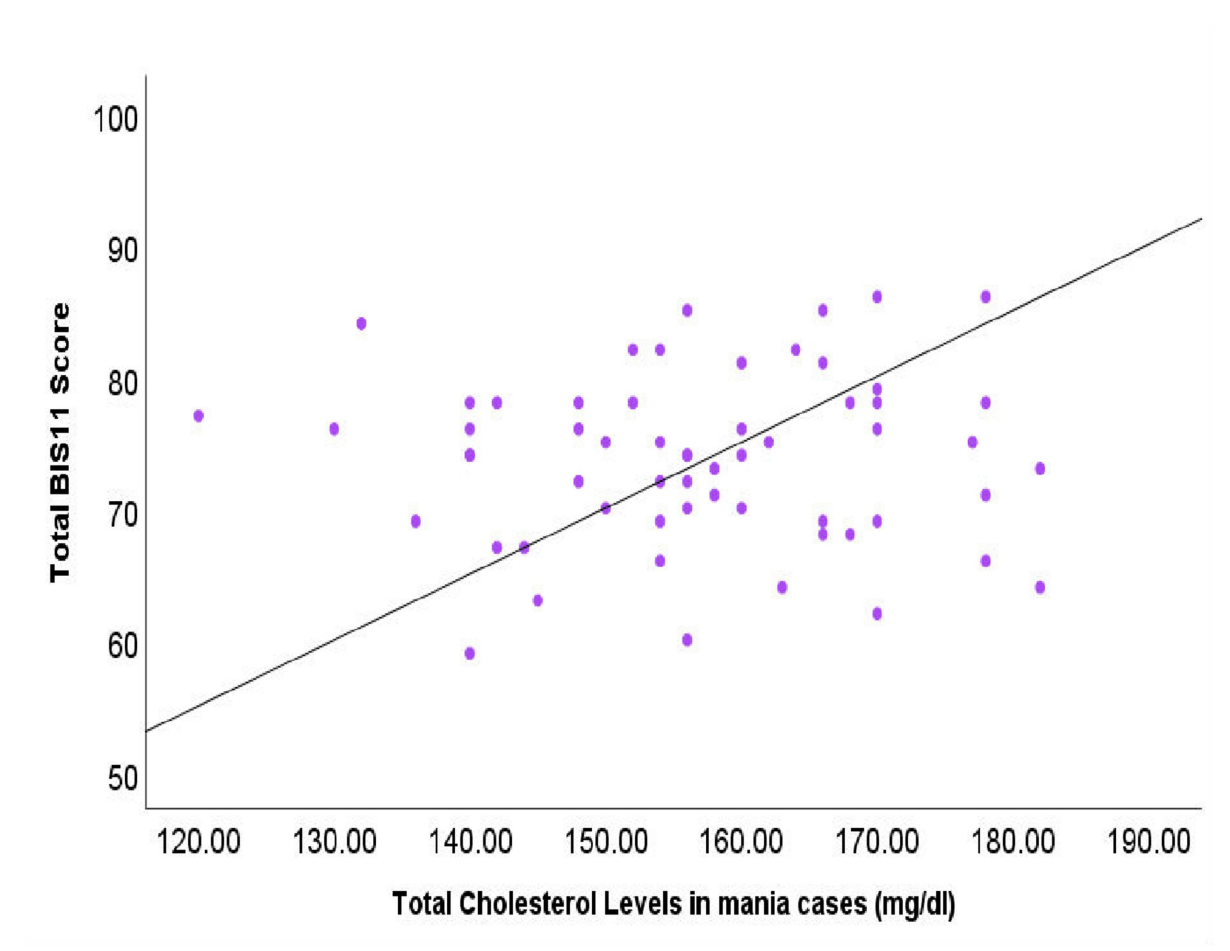
Correlation between total cholesterol levels and impulsivity (BIS-11 scores) in mania patients. BIS-11: Barratt Impulsiveness Scale-11.

**Figure 3:**
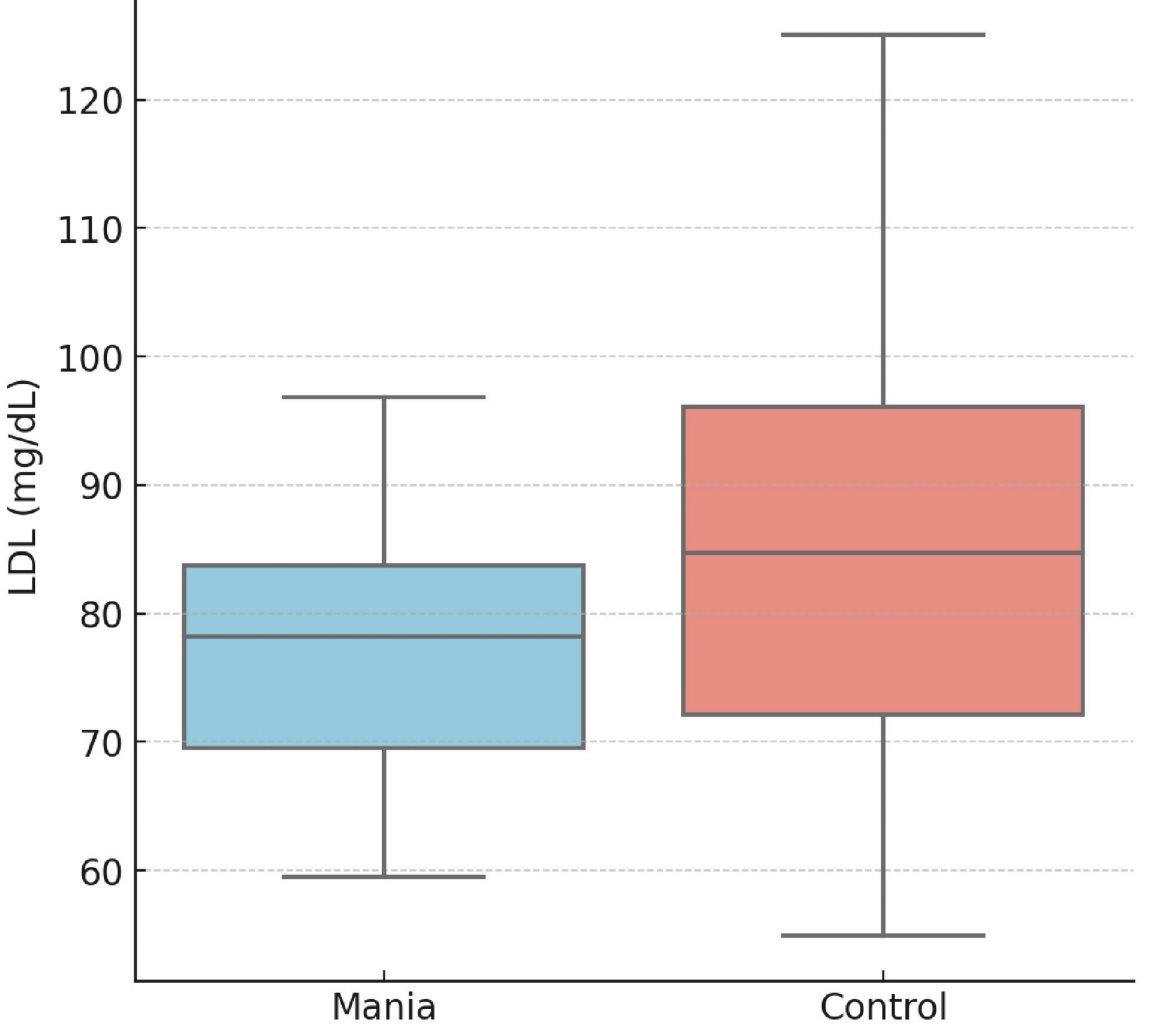
Box-whisker plot comparing LDL levels in mania and control groups. LDL: low-density lipoprotein.

The correlation between lipid profiles and impulsivity, assessed using BIS-11 scores, is presented in Table 3, and the correlation between total cholesterol and impulsivity is presented in Figure 2. A significant negative correlation was observed between impulsivity and total cholesterol (r = -0.42, p < 0.05) as well as LDL levels (r = -0.36, p < 0.05), suggesting that lower lipid levels are associated with higher impulsivity in mania patients. Although triglycerides showed a negative correlation with impulsivity (r = -0.27), this did not reach statistical significance (p = 0.06). HDL (r = 0.12, p = 0.21) exhibited no significant correlation with impulsivity. ApoB demonstrated a trend toward a positive correlation with impulsivity (r = 0.29), but this was not statistically significant (p = 0.07). These findings highlight a potential link between lipid metabolism and impulsive behavior in individuals experiencing manic episodes (Table 3, Figure 3).

**Table 3:** Correlation between lipid profile and impulsivity total score (BIS-11) in first-episode mania patients. *Significant at p-value < 0.05. Pearson correlation coefficient was calculated for the r-value and p-value. LDL: low-density lipoprotein, HDL: high-density lipoprotein, ApoA1: apolipoprotein A1, ApoB: apolipoprotein B, BIS-11: Barratt Impulsiveness Scale-11.

Parameter (unit)	r-value	p-value
Total cholesterol (mg/dL)	-0.42	<0.05*
LDL (mg/dL)	-0.36	<0.05*
Triglycerides (mg/dL)	-0.27	0.06
HDL (mg/dL)	0.12	0.21
ApoA1 (mg/dL)	-0.12	0.498
ApoB (mg/dL)	0.29	0.07

## Discussion

The present study examined the relationship between lipid profile parameters, apolipoproteins, and impulsivity in first-episode manic patients compared to healthy controls. Our findings demonstrate that, although mania patients exhibited significantly lower levels of total cholesterol, triglycerides, LDL, and VLDL compared to controls, there were no significant differences in ApoA1 and ApoB levels. Furthermore, BIS-11 total scores, which reflect overall impulsivity, were significantly and negatively correlated with total cholesterol (r = -0.42, p < 0.05) and LDL (r = -0.36, p < 0.05). A trend towards a positive correlation between Apo B and impulsivity was observed (r = 0.29, p = 0.07), although it did not reach statistical significance.

The data also showed that BMI was significantly higher in mania patients, suggesting that body composition and metabolic factors might interact with lipid metabolism and behavioral regulation in this population [5,14]. However, the significant differences in lipid parameters seem to be independent of the modest BMI differences observed.

These results support previous research suggesting that lower serum cholesterol levels may be associated with increased impulsivity [5,15-17]. The negative correlations between total cholesterol and LDL with impulsivity are consistent with the hypothesis that reduced serum cholesterol may lead to altered central serotonergic transmission. Given that cholesterol is crucial for maintaining membrane fluidity and, consequently, the proper function of serotonin receptors, our findings lend further support to the notion that low cholesterol may impair serotonergic neurotransmission, thereby increasing impulsive behaviors, a phenomenon that has been reported in bipolar disorder [16,17].

While several studies have indicated a relationship between cholesterol fractions and behavioral outcomes, the exact lipid fraction most predictive of impulsivity remains debated [15]. In our study, HDL levels did not significantly correlate with impulsivity, which contrasts with some earlier reports that have implicated HDL as a marker for central serotonergic activity [17-20]. Previous studies have suggested that HDL can prevent the oxidation of LDL. Ezzaher et al. (2010) suggested that patients with mania exhibited significantly higher levels of total cholesterol, LDL-C, ApoB/ApoA1 ratio, and Lp(a), along with significantly lower ApoA1 levels compared to controls, consistent with previous findings linking lipid deregulation to bipolar disorder. The altered lipid profile may be influenced by factors such as nutritional status, reduced physical activity, and medication use, which contribute to metabolic disturbances in these patients [21,22]. Given that cholesterol and other lipoprotein components play essential roles in neuronal membrane integrity and intercellular signaling, these abnormalities may have implications for mood regulation and the neurobiology of mania [22]. Although Apo B is a key structural protein of LDL particles, its non-significant trend towards association with impulsivity may indicate that apolipoproteins could have a subtler role or that larger sample sizes are needed to detect such effects.

Overall, these findings are congruent with the growing body of literature linking hypocholesterolemia to behavioral dysregulation in mood disorders. They highlight the importance of considering serum lipid profiles, especially total cholesterol and LDL levels, as potential biological markers of impulsivity in manic states [23,24]. The observed correlations reinforce the hypothesis that lipid metabolism may contribute to the neurobiological mechanisms underlying impulsivity via alterations in central neurotransmission, particularly in serotonergic circuits. 

Limitations of the study

The present study has a few limitations. The sample size was modest (n = 60), but a bigger sample would have been provided with more reliable findings. Secondly, the study sample had an underrepresentation of the female gender, urban population and subjects from a more socio-economic level background. Again, considering the lifestyle factors like diet and physical activity, which might affect lipid profiles, obesity, and other parameters like the waist-hip ratio, which is a measurement of obesity, were not considered. The study used self-report measure of impulsivity (BIS-11), which depends on individual responses and might have been influenced by the affective states of the subjects. The objective measurements of impulsivity were not done. 

## Conclusions

First-episode mania patients exhibit significantly lower cholesterol levels, which correlate with higher impulsivity scores. Hence, lower lipid levels in bipolar disorder by lipid-lowering drugs and weight loss medication may predict higher impulsivity, which should be clearly monitored to avoid high-risk behavior in these patients. This study therefore adds the growing evidence for complex association between cholesterol level and mental well-being in bipolar disorder to reduce impulsive behavior by lipid-lowering therapies, which are detrimental for bipolar patients. These findings emphasize the need for lipid monitoring in high-risk psychiatric populations and open avenues for future research on lipid-based interventions in bipolar disorder. In summary, the study not only reinforces the link between lipid metabolism and impulsivity but also provides new insights by addressing methodological gaps in the literature and expanding the scope of research to include underrepresented populations and a more diverse lipid profile. Future research should aim to replicate these findings in larger cohorts and explore the mechanistic pathways through which cholesterol and its fractions influence central neurotransmitter systems involved in mood regulation and impulsivity.
